# Comprehensive panicle phenotyping reveals that ***qSrn7/FZP*** influences higher-order branching

**DOI:** 10.1038/s41598-018-30395-9

**Published:** 2018-08-21

**Authors:** Yasuko Fujishiro, Ayumi Agata, Sadayuki Ota, Ryota Ishihara, Yasumi Takeda, Takeshi Kunishima, Mayuko Ikeda, Junko Kyozuka, Tokunori Hobo, Hidemi Kitano

**Affiliations:** 1 Graduate School of Bioagricultural Sciences, Nagoya University, Furo, Chikusa, Nagoya, Aichi 464-8601 Japan; 2 Bioscience and Biotechnology Center, Nagoya University, Furo, Chikusa, Nagoya, Aichi 464-8601 Japan; 3 Graduate School of Life Sciences, Tohoku University, Katahira, Aoba, Sendai, Miyagi 980-8577 Japan

## Abstract

Rice grain number directly affects crop yield. Identifying alleles that improve panicle architecture would greatly aid the development of high-yield varieties. Here, we show that the quantitative trait locus *qSrn7* contains rice *FRIZZY PANICLE* (*FZP*), a previously reported gene encoding an ERF transcription factor that promotes floral transition. Reduced expression of *FZP* in the reproductive stage increases the extent of higher order branching of the panicle, resulting in increased grain number. Genotype analysis of this gene in cultivars from the publicly available National Institute of Agrobiological Sciences (NIAS) Core Collection demonstrated that the extent of higher order branching, especially in the upper panicle, was increased in those cultivars carrying the *FZP* allele associated with *qSrn7*. Furthermore, chromosome segment substitution lines resulting from a cross between Koshihikari and Kasalath, the latter of which carries *qSrn7/FZP*, also showed that upper panicle higher order branching and grain yield were increased by *qSrn7/FZP*. Our findings indicate that *qSrn7/FZP* influences panicle branching pattern and is thus useful in the breeding of high-yield rice varieties.

## Introduction

**Figure 1:**
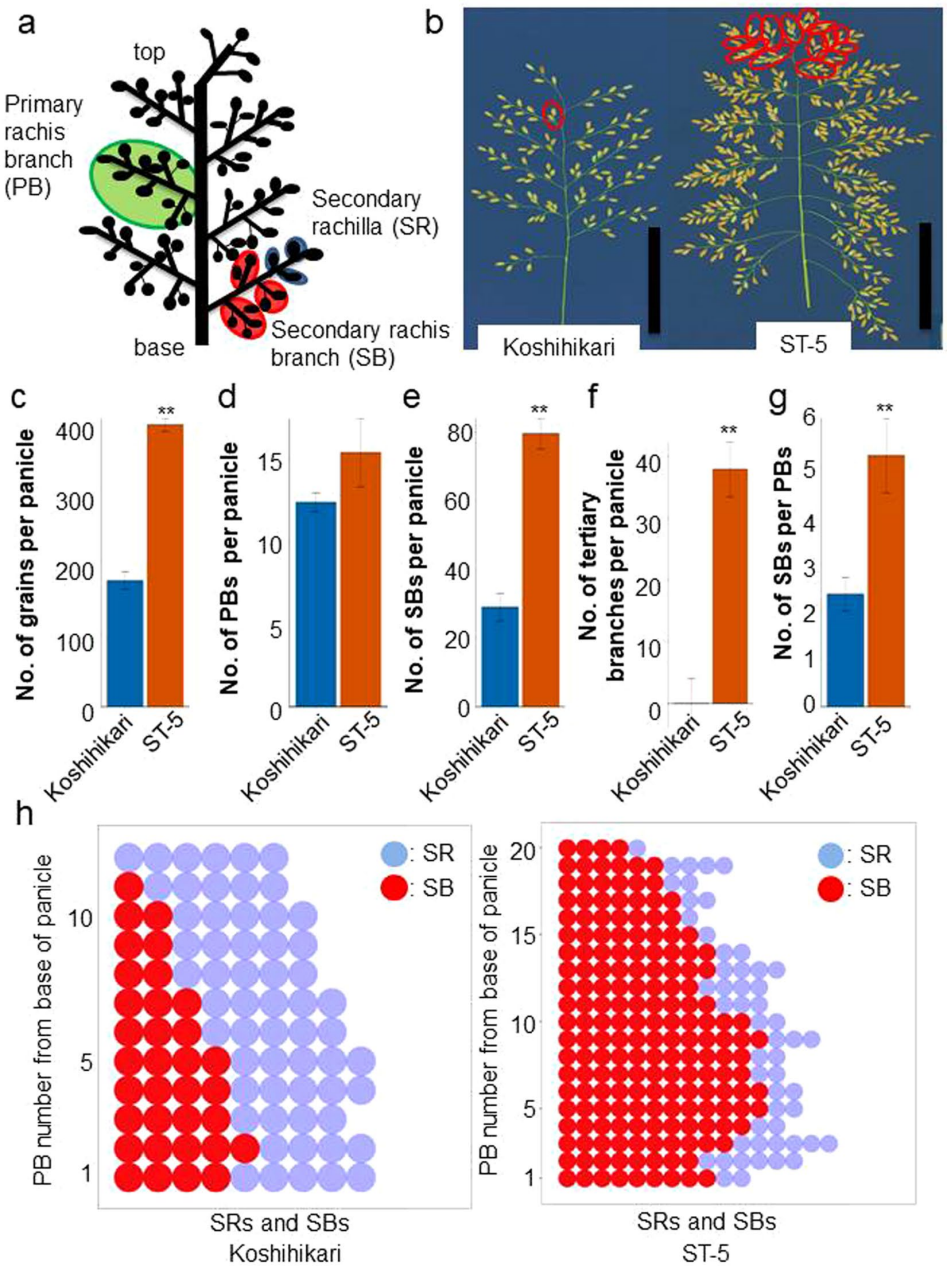
Panicle branching pattern of Koshihikari and ST-5. (**a**) Schematic of a rice inflorescence. (**b**) Panicle morphologies of Koshihikari and ST-5. Scale bar: 10 cm. Red circles indicate secondary rachis branches (SBs). (**c–g)** Comparison of panicle attributes between Koshihikari and ST-5. (**c**) Number of grains per main panicle. (**d**) Number of primary rachis branches (PBs) per main panicle. (**e**) Number of SBs per main panicle. (**f**) Number of tertiary branches per main panicle. (**g**) Comparison of number of SBs per PBs between Koshihikari and ST-5. (**h**) Panicle branching pattern of Koshihikari and ST-5. Red circles: SBs. Blue circles: Secondary rachillae (SRs). **Significant at 1% level (Student’s *t*-test).

Food security has become a pressing global problem because of an increasing world population and climate change^1,2^. Rice (*Oryza sativa* L.) is a vital crop worldwide and sustains more than three billion people^3^. The panicle is a pivotal organ in determining rice grain yield, and its size and architecture are key targets in selective breeding for improved rice grain yield and quality^4,5^. The rice panicle is comprised of several organs, namely the rachis (the main axis), primary rachis branches (PBs), and secondary rachis branches (SBs), which include additional higher-order rachis branches (Fig. 1a). Panicle branching pattern is based on the development of SBs on each PBs and it determines panicle structure as well as final grain number and ultimately grain yield^5,6^.

Many genes have been identified that are associated with rice panicle development. Through mutant characterization, it was shown that *apo1* (*aberrant panicle organization1*) reduces panicle development, resulting in fewer panicle rachis branches^7,8^, and *lax* (*lax panicle*) inhibits rachis branching^9,10^. Although these mutants are useful for elucidating the regulatory mechanisms of panicle development, their applicability in agriculture is limited because they have abnormal plant architecture. By contrast, quantitative traits found in various rice cultivars are influenced by multiple alleles derived from the accumulation of natural mutations. Several of these alleles are associated with grain yield. A natural allele of *DENSE AND ERECT PANICLE 1* (*DEP1*) was shown to influence panicle architecture, resulting in reduced panicle internode length and increased grain number^11^, whereas *Grain Number 1a* (*GN1a*)^12^ and *WEALTHY FARMER’S PANICLE* (*WFP*)^13^ were shown to significantly increase PB number, grain number, and yield. These past studies have examined terminal phenotypes such as grain number and panicle length; however, panicle structure as determined by its branching pattern has not been characterized in detail, such as through comprehensive analysis of several organs.

In the rice panicle, flower opening and grain filling occur in a sequential developmental process, which is initiated in the spikelet furthest from the rachis axis and proceeds inward. As a result, the position of a spikelet within a panicle determines the timing of both flower fertilization and grain development. Panicle size and branching pattern are thought to determine grain shape and quality directly^14^. High-yield modern rice varieties have higher grain filling rate in upper areas of the panicle^15,16^. The rice panicle exhibits a recursive structure in which higher-order rachis branches radiate from lower-order rachis branches, which in turn radiate from the rachis. Therefore, knowledge of how the order of each rachis branch is affected by a particular gene will greatly assist in efforts to alter the architecture of the rachis for optimal yield. Furthermore, identifying a novel quantitative trait locus (QTL) that regulates these organs would assist in breeding new rice varieties with increased yields.

Here, we describe the identification and characterization of a major QTL, *qSrn7*, which regulates a novel panicle branching pattern in rice. Analysis of chromosome segment substitution lines (CSSLs) carrying *qSrn7* showed that grain number and yield increased in conjunction with changing panicle structure and branching pattern. These results describe the mechanism by which *qSrn7* influences panicle structure, and suggest that *qSrn7* may be used in efforts to generate rice varieties with high grain yield.

## Results

### Panicle morphology and branching pattern

To identify genes that influence rice grain yield, we selected two rice varieties that display clear differences in grain number per panicle, namely Koshihikari, a typical Japonica rice variety, and ST-5, from the Stocked rice collections of Togo field and Nagoya University-5. Koshihikari and ST-5 have approximately 182 and 394 grains in the main panicle, respectively (Fig. 1b,c). The panicle of ST-5 is larger than that of Koshihikari (Fig. 1b–f).

To define the panicle structure that results in the increased grain number of ST-5, we schematically described the panicle branching pattern by detailing the number of secondary rachillae (SRs) and SBs on each PB (Fig. 1h). Each PB of ST-5 contained more than twice as many SBs as the equivalent PB of Koshihikari (Fig. 1g). In particular, the proliferation of SBs was greater in the upper areas of ST-5 panicles than in those of Koshihikari (Fig. 1h).

### Isolation and characterization of *qSrn7*

**Figure 2:**
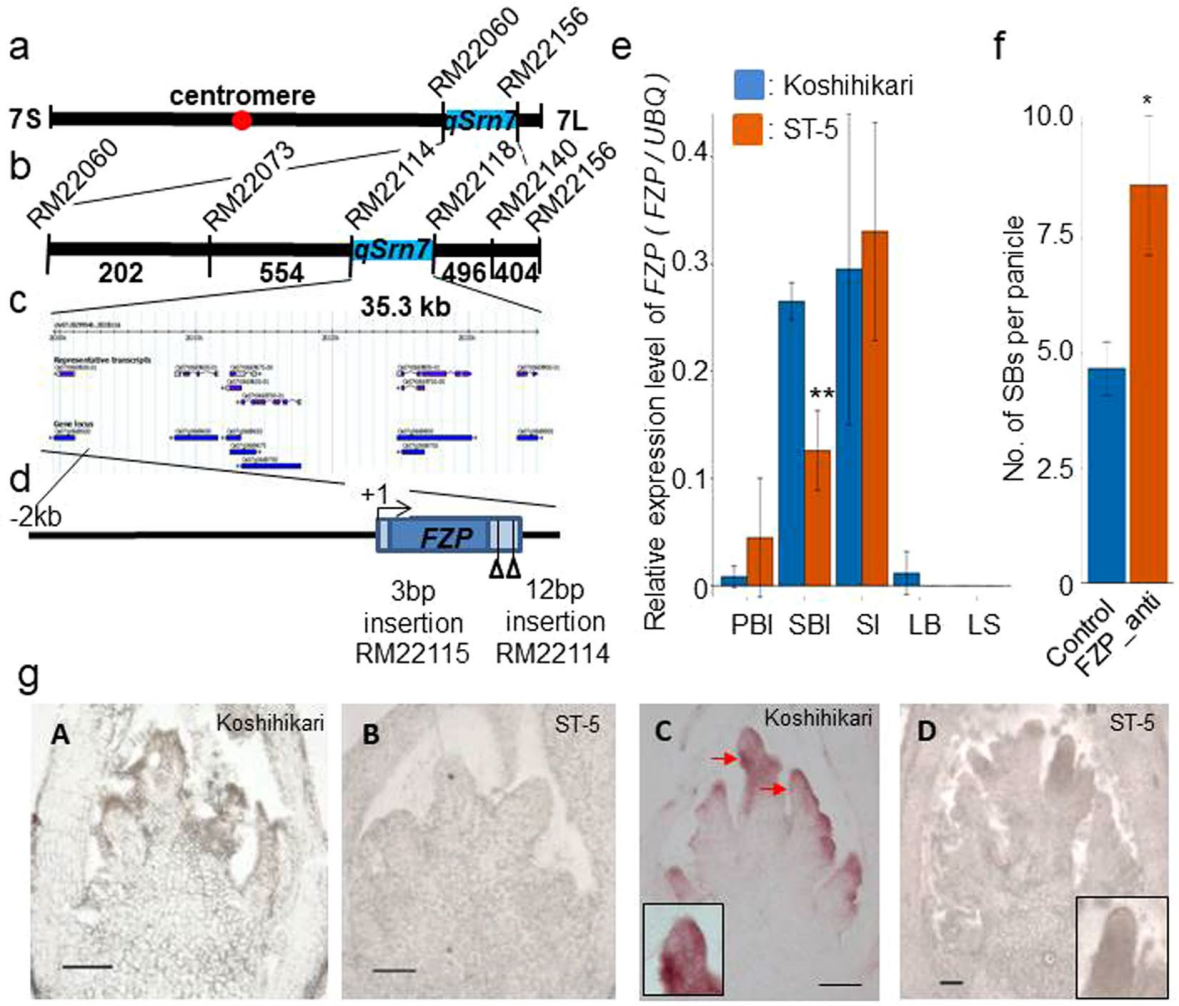
Aanalysis of *FZP* expression and *FZP* antisense transgenic phenotype. (**a**–**d**) Fine mapping of *qSrn7*. (**a**) The *qSrn7* locus was detected between RM22060 and RM22156 on Chromosome 7. (**b**) Numbers on the map indicate the number of recombinants. (**c**) The candidate region of *qSrn7* was located to the 35.3-kb region between RM22114 and RM22118 by fine mapping. (**d**) The *FZP* locus. Nucleotide insertions present in ST-5 are indicated. (**e**) *FZP* expression analysis in inflorescence tissue at various developmental stages and in leaf tissue by quantitative real-time PCR. PBI: Primary rachis branch initiation stage, SBI: Secondary branch initiation stage, SI: Spikelet initiation stage of inflorescence development. LB: Leaf blade, LS: Leaf sheath of leaf. (**f**) Comparison of number of SBs per main panicle between vector control and *FZP* antisense transgenic plants. (**g**) *In situ* hybridization of *FZP* transcript during panicle development in Koshihikari (A,C) and ST-5 (B,D). (A–D) Developing inflorescence at the stage of primary (A,B) and secondary (C,D) branch differentiation. (Insets) Close-up view of the apical region of a developing primary branch that is initiating a secondary rachis branch meristem. Arrowheads indicate incidence region of expression. Scale bars: 100 μm. **Significant at 1% level. *Significant at 5% level (Student’s *t*-test).

To identify the gene responsible for the increased number of SBs and thus the increased grain number in ST-5 panicles, we performed a QTL analysis using an F_2_ population derived from a cross between Koshihikari and ST-5. We detected one major QTL with a log_10_ odds (LOD) score of >3 on chromosome 7 (LOD score = 13.6), which was denoted as *qSrn7* (QTL for secondary rachis branching on chromosome 7). A total of 1700 F_3_ individuals derived from heterozygous Koshihikari and ST-5 F_2_ plants were screened for recombination within the target region defined by simple sequence repeat (SSR) markers RM22060 and RM22156 (Fig. 2a). High-resolution linkage mapping analysis located *qSrn7* to a 35.3 kb-region between SSR markers RM22114 and RM22118 (Fig. 2b). According to the Rice Annotation Project Database (http://rapdb.dna.affrc.go.jp/), there are eight predicted open reading frames (ORFs) in this region (Fig. 2c), including Os07g0669500, known as *FRIZZY PANICLE* (*FZP*), which encodes an AP2/ERF-type transcription factor that regulates spikelet number in the panicle^17,18^. We hypothesized that the phenotype of increased SBs in ST-5 panicles was caused by weak *FZP* activity. Therefore, we performed an allelism test between ST-5 and *fzp* plants. The panicles of the resulting F_1_ hybrids displayed a ST-5 panicle phenotype, indicating that genetic complementation of *fzp* had occurred (Supplementary Fig. 1). Comparative sequence analysis of *FZP* in Koshihikari and ST-5 revealed that the latter allele, herein referred to as *qSrn7/FZP*, contained insertions in the 3′ UTR (Fig. 2d).

Expression analysis by quantitative real-time PCR revealed *FZP* expression in three inflorescence developmental stages associated with branching initiation, and no *FZP* expression in leaf tissue (Fig. 2e). Of note, *qSrn7/FZP* expression was lower in the SB initiation stage in ST-5 than in Koshihikari at the inflorescence developmental stage. We also determined the temporal and spatial expression patterns of *FZP* by *in situ* hybridization analyses in various inflorescence developmental stages (Fig. 2g). *FZP* expression was detected in Koshihikari close to the spikelet meristems in the SB initiation stage (Fig. 2g, Panel C), whereas only slight *qSrn7/FZP* expression was detected in equivalent ST-5 tissue (Fig. 2g, Panel D).

To confirm that lower levels of *qSrn7/FZP* transcript are responsible for the associated panicle branching phenotype, a *FZP* antisense DNA fragment was introduced into the genome of the Japonica variety Nipponbare through *Agrobacterium*-mediated transformation. Several of the resulting *FZP* antisense transgenic plants had more SBs than the vector control plants, indicating that *FZP* regulates SB number (Fig. 2f).

### Effects of *qSrn7/FZP* on panicle branching pattern

**Figure 3:**
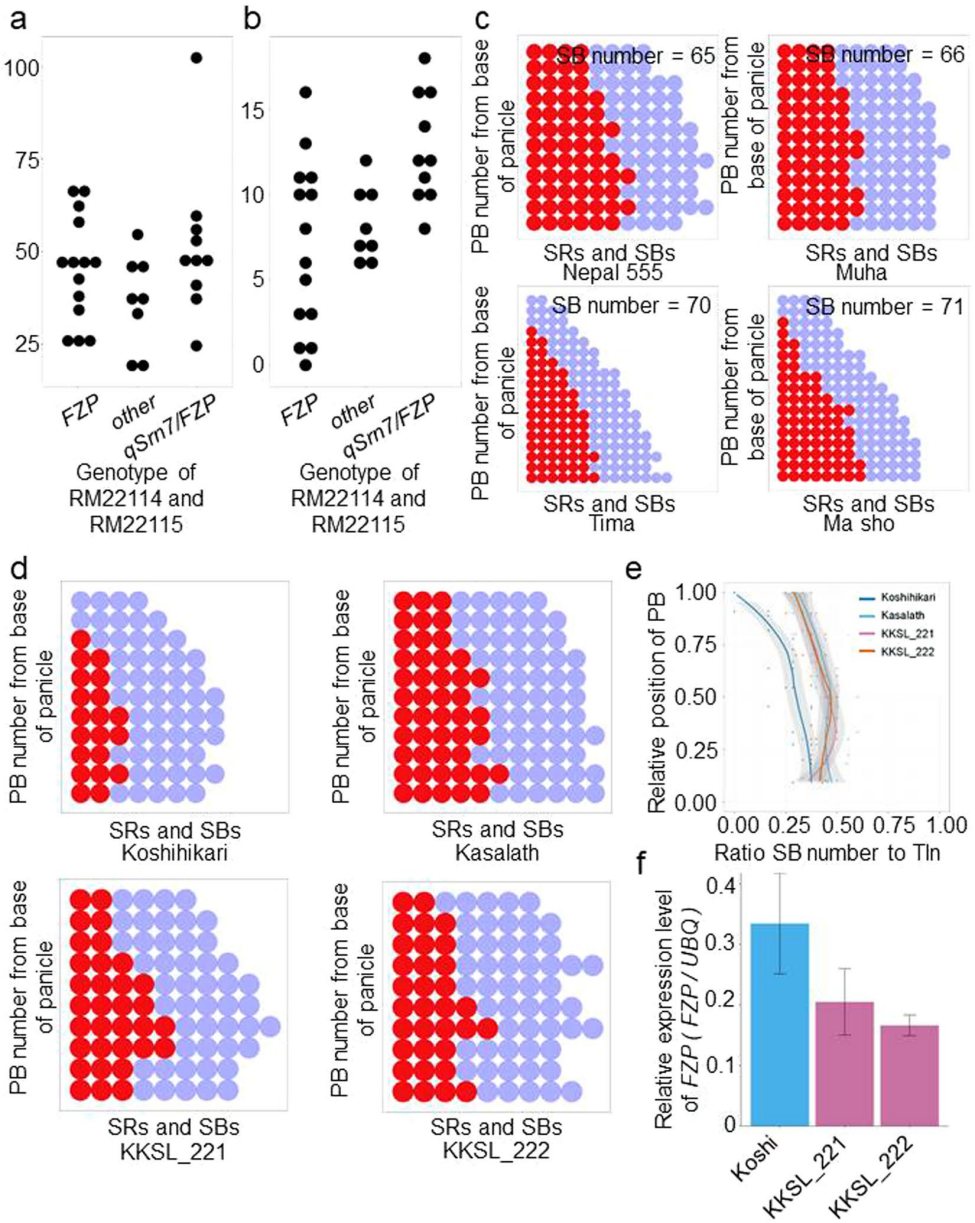
Panicle branching pattern. (**a**) Dot plots of total SB number per panicle against the genotype determined at RM22114 and RM22115. (**b**) Dot plots of SB number per upper three PBs of the panicle against the genotype determined at RM22114 and RM22115. (**c**) Panicle branching patterns in lines carrying ST-5 (top panels) and Koshihikari (bottom panels) genotypes determined at RM22114 and RM22115. (**d**) Panicle branching pattern of Koshihikari, Kasalath, and two CSSLs (KKSL_221 and KKSL_222). PB: primary rachis branch. Red circles: secondary rachis branches (SBs). Blue circles: secondary rachillae (SRs). (**e**) Comparison of panicle branching patterns of Koshihikari, Kasalath, and CSSLs. Tln: Total number of lateral branches on the PB (SBs + SRs). Solid lines denote the loess smoothing curves and shadows represent the estimated SE. (**f**) Relative expression of *FZP* in Koshihikari and CSSLs in developing inflorescences at the secondary branch initiation stage.

To investigate the functional presence of *qSrn7/FZP* in other rice cultivars, we genotyped the *FZP* 3′ UTR of 30 cultivars, including the varieties Japonica and Indica, using SSR markers RM22114 and RM22115 (Supplementary table 1). The resulting genotypes were then compared with SB number and branching pattern. There was no apparent difference in total SB number per panicle between lines carrying either *FZP* or *qSrn7/FZP* (Fig. 3a). However, for the upper three PBs of the panicle, lines carrying *qSrn7/FZP* had slightly more SBs than did lines carrying *FZP* (Fig. 3b). Furthermore, analysis of panicle branching pattern showed that SB number in the upper regions of the panicle was considerably higher in lines carrying *qSrn7/FZP* than in lines carrying *FZP*, although total SB number per panicle was lower in lines carrying *qSrn7/FZP* (Fig. 3c).

To further evaluate the effect of *qSrn7/FZP* on SB number in the upper regions of the panicle, we analyzed CSSLs^19^ created from a cross performed between Koshihikari (as a recurrent parent) and Kasalath carrying *qSrn7/FZP*. The progeny of the cross are herein referred to as KKSLs. SB number in the upper regions of the panicle was higher in Kasalath and the KKSLs (KKSL_221 and KKSL_222) than in Koshihikari (Fig. 3d). This increase in higher-order branching was represented by the ratio of SB number to lateral branch number (Tln; SB number + SR number) on PBs in Kasalath, Koshihikari, and the KKSLs. In Kasalath and the KKSLs, the ratio of SB number to Tln was >0.25. Interestingly, the Local Regression (loess) curves of the KKSLs were similar to those of Kasalath, but the ratio of SB number to Tln decreased in Koshihikari in upper regions of the panicle (Fig. 3e). Expression of *qSrn7/FZP* in the KKSLs was approximately half the level of *FZP* expression in Koshihikari at the SB initiation stage (Fig. 3f). These results suggest that the *qSrn7/FZP* allele has reduced expression in tissue at the SB initiation stage, which causes an increase in higher order branching in upper regions of the panicle by preventing the transition from branch to spikelet meristem, as seen in the KKSLs.

### Effects of *qSrn7/FZP* on higher order branching pattern and increased grain productivity

**Figure 4:**
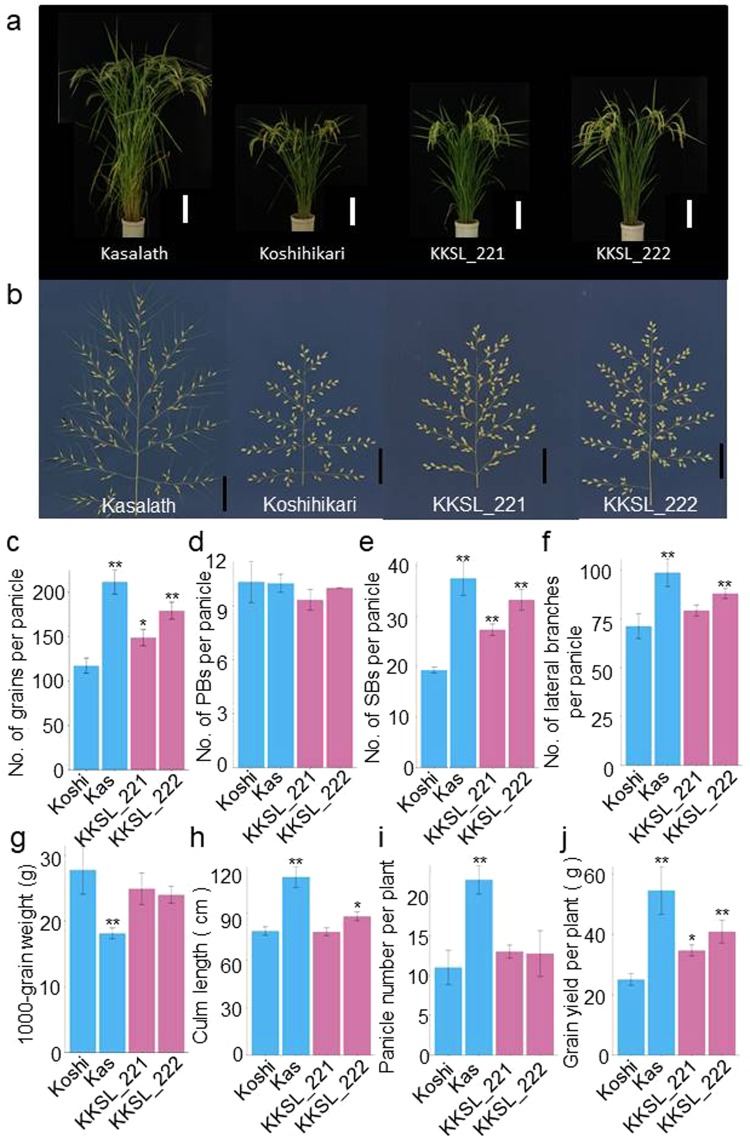
Grain yield performance of CSSLs compared with Koshihikari. (**a**) Plant morphology of Koshihikari, Kasalath, and CSSLs at the grain ripening stage. Scale bar: 20 cm. (**b**) The panicle morphologies of Kasalath, Koshihikari, and the CSSL KKSL_222. Scale bar: 5 cm. (**c–g**) Comparison of main panicle traits between Koshihikari, Kasalath, and two CSSLs. (**c**) Grain number per panicle. (**d**) Number of primary rachis branches (PBs) per panicle. (**e**) Number of secondary rachis branches (SBs) per panicle. (**f**) Total number of lateral branches (secondary rachillae (SRs) + SBs) per panicle. (**g**) The 1000-grain weight. (**h–j**) Comparison of yield factors between Koshihikari, Kasalath, and two CSSLs. (**h**) Culm length. (**i**) Panicle number per plant. (**j**) Grain yield per plant. Blue bars: parental lines of CSSLs. Red bars: CSSLs. **P < 0.01; *P < 0.05 versus Koshihikari (Dunnett’s multiple comparison test).

As *qSrn7/FZP* was found to affect higher order branching in upper regions of the panicle, we evaluated the effect of *qSrn7/FZP* on rice grain yield. At first glance, the KKSLs displayed a similar plant and panicle morphology to Koshihikari (Fig. 4a,b). However, total grain number per plant in the KKSLs was higher than that in Koshihikari (Fig. 4c). These differences in grain number depend mainly on the extent of higher order branching in the panicle (Figs 3d,e and 4e). SB number per panicle in the KKSLs was approximately 40% higher than that in Koshihikari (Fig. 4e), whereas PB number was similar in Koshihikari and the KKSLs (Fig. 4d). Consequently, *qSrn7/FZP* increased the total grain number in the KKSLs by 30–50% (Fig. 4c), which was caused primarily by an increase in SB number in the upper regions of the panicle (Figs 3d and 4c). No differences were observed between Koshihikari and the KKSLs for other factors contributing to grain yield, such as PB number (Fig. 4d), panicle number per plant (Fig. 4i), and 1000-grain weight (Fig. 4g). As anticipated, the grain yield per plant in the KKSLs was 40–60% higher than in Koshihikari (P < 0.05; Fig. 4j). These results suggest that *qSrn7/FZP* affects grain yield.

## Discussion

Panicle architecture is the major target in efforts to improve crop yield in rice. *GN1*, a major QTL for grain number, is associated with panicle architecture and has already been incorporated into breeding programs. However, the function of *GN1* was characterized based on its effect on terminal phenotypes, such as grain number, which does not consider panicle branching pattern and panicle structure. Quantifying panicle traits tends to be laborious and time-consuming because of the large number of complex traits; however, several recent studies have been based on QTL or GWAS analyses using phenotype data collected with high-resolution panicle phenotyping software^6,20^. Therefore, to our knowledge, further genes involved in panicle architecture have also not been thoroughly examined for their effects on panicle branching pattern using panicle trait analysis. In 1989, the International Rice Research Institute (IRRI) launched a breeding program for New Plant Type (NPT) rice to increase the yields of modern indica cultivars by incorporating genetic material from tropical japonica landraces^21^. The NPT breeding program attempted to boost yield by simultaneously selecting for increased panicle size (sink) and photosynthetic capacity (source)^21^. The NPT cultivar successfully generated large panicle phenotypes but had lower yields than modern indica cultivars, because it did not exhibit the desired combinations of sink and source traits that are characteristic of high-yielding varieties^22,23^. This result suggests that physiologically optimizing panicle architecture for grain filling and yield will probably involve managing a highly interactive network or trait complex. Thus, analyses that consider complex panicle phenotypes are of great importance^20^. Furthermore, as changes to panicle branching pattern and structure are associated with increases in grain yield^15^, it is important to characterize how a particular gene affects the panicle branching pattern.

Efforts to improve panicle grain number would benefit both from focusing on previously characterized genes that influence branching pattern and on identifying additional genes that influence panicle traits. To the best of our knowledge, genes that affect panicle branching pattern had not hitherto been identified. Therefore, we used a rice variety with increased panicle grain number to identify and characterize a gene involved in panicle branching pattern.

Analysis of the QTL *qSrn7* revealed that the locus contains *FZP* and its maize ortholog *BRANCHED SILKLESS 1* (*BD1*)^24^, which encodes an AP2/ERF-type transcription factor. Furthermore, we showed that *qSrn7* in the existing variety ST-5 and in CSSLs using in this study (KKSLs carrying *qSrn7* from Kasalath) produced more SBs with attached filled grains (Fig. 4g), and that the *FZP* expression level in spikelet meristems of these lines was slightly reduced (Fig. 3f). Our results suggest that *qSrn7* is a weakly active allele of *FZP*, which we denoted as *qSrn7/FZP*. Although *fzp* rice lines, which are devoid of FZP activity, develop more secondary and high-order panicle branches, they produce fewer filled grains^17^. Recent studies have indicated that *FZP*-overexpressing plants accelerate the transition between spikelet and floral meristem, which results in a short panicle with fewer branches^18^. These results support a negative regulatory role for *FZP* in panicle branching. Sequence comparison suggests that the 3′ UTR of *FZP* may contribute to the reduced *qSrn7*/*FZP* expression level observed in this study, although the exact molecular mechanism for this is unclear. Previous studies have indicated that post-transcriptional control, including mRNA stability and translation, plays a crucial role in plant growth and development. The length of the 3′ UTR is sensed in the process of mRNA quality control^25,26^. In this study, the 3′ UTR of *qSrn7*/*FZP* was found to be longer than that of *FZP* (Fig. 2d). Therefore, the long 3′ UTR of *qSrn7*/*FZP* may be responsible for the low *qSrn7*/*FZP* expression levels that result in an increased number of SBs. Furthermore, rice *qSrn7*/*FZP* cultivars were observed to have more SBs than the *FZP* cultivars on the upper PBs of the panicle (Fig. 3e,f), suggesting that rice *qSrn7*/*FZP* cultivars may benefit rice breeding projects aimed at improving grain yield.

Panicle architecture is an important target for rice crop improvement, and genes associated with panicle architecture, such as *DEP1*, *GN1*, and *WFP*, have already proven to be useful in rice breeding programs^11–13^. However, little is known about how these genes affect panicle branching pattern. Matsuba^27^ reported that the panicle structure of rice cultivars should be classified into five panicle branching patterns based on the differential ability of SBs to form on each PB during developmental process. The panicles of a number of high-yield rice cultivars were classified into five panicle branching patterns^6^. These findings suggest that the panicles of individual high-yield rice cultivars possess different panicle branching patterns, which suggests that various desirable panicle branching phenotypes may be combined in future breeding strategies to develop varieties with panicles that produce even more grain. In this study, we demonstrated that a QTL designated as *qSrn7* effectively increases SB number, but does not influence PB number. The generated KKSLs carrying *qSrn7* displayed a gradual increase in SB number in the upper regions of the panicle (Fig. 3d) through enhanced branching of the lateral meristem (Fig. 3e), which increased grain yield. These results show that *qSrn7/FZP* affects higher order branching, and suggests that combining *qSrn7/FZP* with other genes affecting panicle structure would be a beneficial strategy in projects aimed at developing novel high-yield rice varieties. Furthermore, this study demonstrates that the combination of QTL analysis using natural variation and the detailed analysis of panicle branching pattern is a useful strategy for identifying genes that can further increase crop productivity.

## Materials and Methods

### Plant materials and growth conditions

Experimental plant material was grown in the research field of Togo Field for Science and Education at Nagoya University, Japan. Field experiments for the grain yield were performed for two years, in 2015–2016. We observed similar results for two years, therefore the cultivation results for 2015 are indicated here. The transgenic plants were grown in isolated greenhouses under standard growth conditions.

### Observation of panicle branching pattern

Main panicle per plant was used for analysis of panicle branching pattern. Measured parameters were the number of grains, PBs, SBs, tertiary branches; the position of PB, SR and SB.

### QTL analysis

QTL analysis was performed with 94 F_2_ plants and the software package R/QTL (R version 3.1.3; R/qtl package 1.31–5). QTLs were identified using Haley–Knott regression and the significance threshold was set using 1,000 permutations. SSR markers used in positional cloning are listed in Supplementary Table 2.

### Vector construction and transformation

For antisense expression of *FZP*, the coding sequence of *FZP* was amplified from Koshihikari cDNA using a forward primer (5′-GCAAGCTTTCAATGGGAGAGGAAGCTGAATGG-3′) containing a HindIII site and a reverse primer (5′-GCACTAGTATGAACACTCGAGGCAGCGGCAGT-3′) containing a SpeI site. The fragment was cloned into the HindIII–SpeI sites of pUbi::nos-pCAMBIA1380 and introduced into Nipponbare, a japonica variety, by *Agrobacterium tumefaciens* (EHA105)-mediated transformation according to the methods of Ozawa *et al*.^28^. Transformed cells and plants were selected by hygromycin resistance, and regenerated seedlings were grown to maturity in pots under greenhouse conditions. More than ten independent T_0_ plants were isolated and five plants were analyzed. As a control, transformants containing an empty vector were included, and five of the resulting control plants were included in each analysis.

### RNA isolation and gene expression analysis

Total RNA from various organs was prepared as described by Sambrook *et al*.^29^ with some modifications and was treated with DNase I. First-strand complementary DNA (cDNA) was synthesized from 1 μg of total RNA using a QuantiTect Reverse Transcription Kit (Qiagen). Quantitative real-time PCR was performed using the QuantiTect SYBR Green PCR Kit (Qiagen) and a LightCycler System (LightCycler 1.5; Roche Applied Science). Transcript levels were measured in three independent biological replicates. The *UBQ* gene from rice was used as an internal standard for normalizing variations in cDNA concentration. The primer sets used for PCR were as follows: FZP primers, 5′-CACATTGGCTCGTACGGTC-3′ and 5′-GAGAAGAGGAAGTCGTGG-3′; UBQ primers, 5′-AGAAGGAGTCCACCCTCCACC-3′ and 5′-GCATCCAGCACAGTAAAACACG-3′.

### Localization of *qSrn7/FZP* transcript by *in situ* hybridization

Tissues were fixed with 4% (w/v) paraformaldehyde and 0.25% glutaraldehyde in 0.1 M sodium phosphate buffer (pH 7.2) overnight at 4 °C and embedded in SCEM compound (SECTION-LAB, Japan). The cut surface was covered with an adhesive film (Cryofilm type IIC9, SECTION-LAB, Japan) and frozen sections (8–14 μm) were prepared with a cryostat (CM 1850 Leica Microsystems, Germany) according to a previously described method^30^. Digoxigenin-labeled antisense RNA probes for *qSrn7/FZP* transcript were prepared as described by Komatsu *et al*.^17^. Hybridization and immunological detection of the digoxigenin-labeled probes were performed according to a previously described method^31^ with some modifications.

## Electronic supplementary material

Dataset 1
