# Supplementary material for: Comprehensive panicle phenotyping reveals that qSrn7/FZP influences higher-order branching
Source: Sci Rep. 2018 Aug 21;8:12511. doi: 10.1038/s41598-018-30395-9 (PMC6104091; doi:10.1038/s41598-018-30395-9)
Supplement: Supplementary file 1 — Dataset 1 [file 41598_2018_30395_MOESM1_ESM.doc]

**Supporting Information**

**Comprehensive panicle phenotyping reveals that *qSrn7/FZP* influences higher-order branching**

Yasuko Fujishiro^1*^, Ayumi Agata^1*^, Sadayuki Ota^1*^, Ryota Ishihara^1^, Yasumi Takeda^1^, Takeshi Kunishima^1^, Mayuko Ikeda^2^, Junko Kyozuka^3^, Tokunori Hobo^2*^, Hidemi Kitano^2*^

^1^Graduate School of Bioagricultural Sciences, Nagoya University, Furo, Chikusa, Nagoya, Aichi 464-8601, Japan

^2^Bioscience and Biotechnology Center, Nagoya University, Furo, Chikusa, Nagoya, Aichi 464-8601, Japan

^3^Graduate School of Life Sciences, Tohoku University, Katahira, Aoba, Sendai, Miyagi 980-8577, Japan

* These authors contributed equally to this work.

***Corresponding author:** Tokunori Hobo and Hidemi Kitano

**
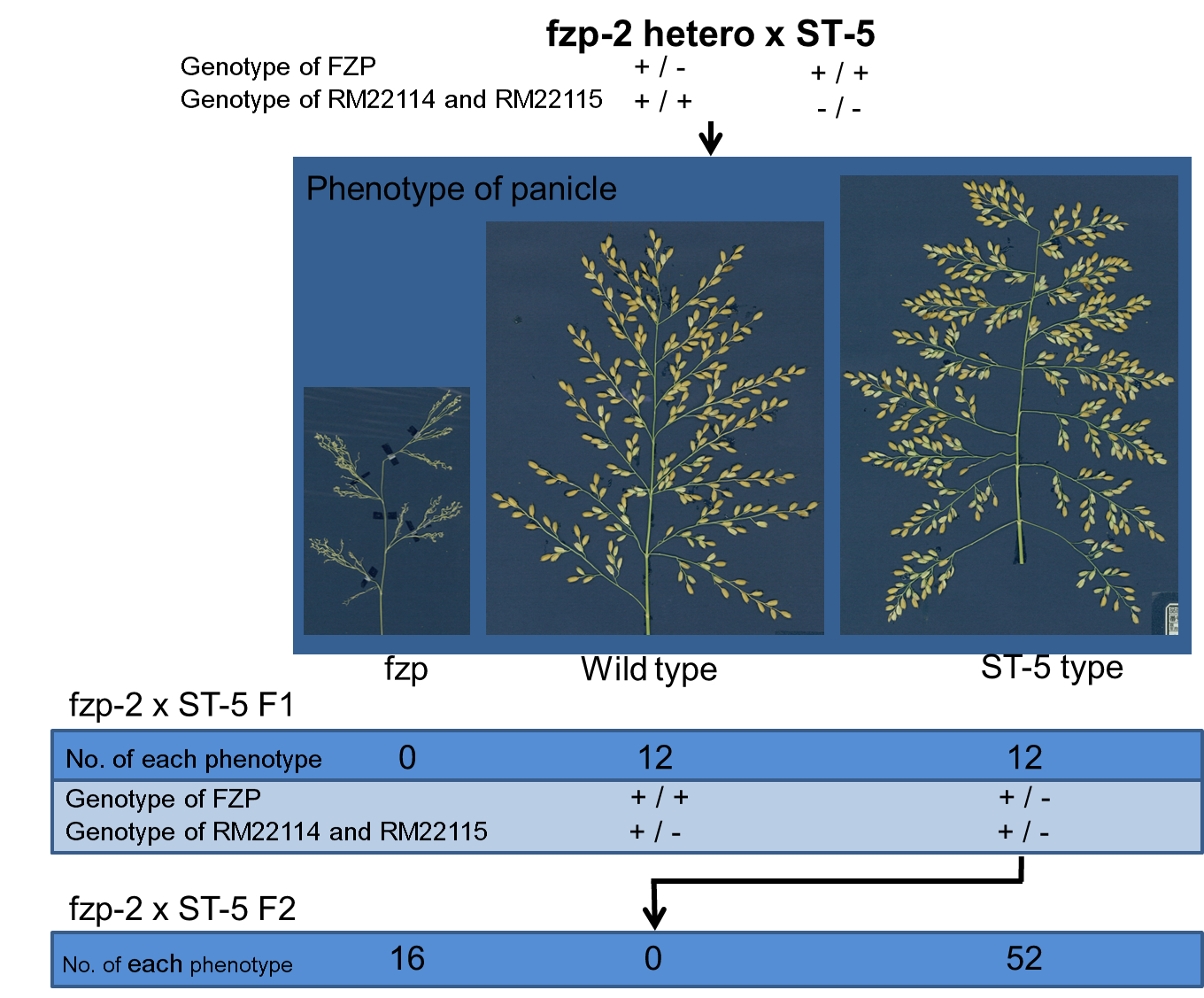
**

**Supplementary Figure 1. Test of allelism between fzp-2 allele.**

**
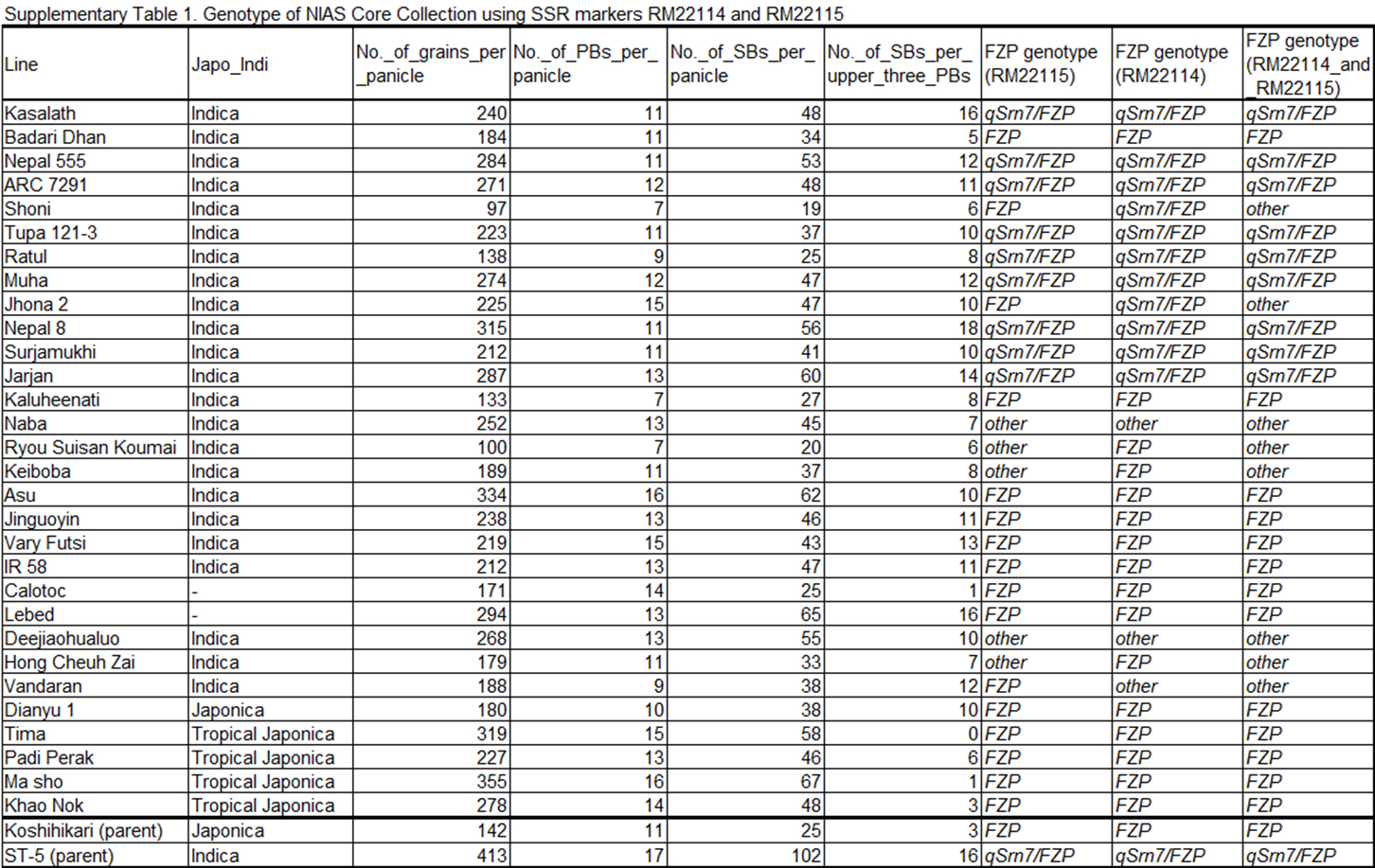
**
